# Diabetes and long duration leading to speech-, low/mid-, and high- frequency hearing loss: current evidence from the China National Health Survey 2023

**DOI:** 10.1007/s40618-024-02406-2

**Published:** 2024-06-13

**Authors:** H. Huang, Y. Fan, F. Yan, Y. Hu, H. He, T. Xu, X. Zhu, Y. Zhu, W. Diao, X. Xia, J. Tu, A. Li, B. Lin, Q. Liu, Z. Lu, T. Xi, W. Wang, D. Xu, Z. Chen, Z. Wang, X. Chen, G. Shan

**Affiliations:** 1 Department of Epidemiology and Statistics, Institute of Basic Medical Sciences Chinese Academy of Medical Sciences, School of Basic Medicine Peking, Union Medical College, Beijing, China; 2 State Key Laboratory of Common Mechanism Research for Major Diseases, Beijing, China; 3 Department of Otolaryngology-Head and Neck Surgery, State Key Laboratory of Complex Severe and Rare Diseases, Peking Union Medical College Hospital, Peking Union Medical College and Chinese Academy of Medical Sciences, Beijing, China; 4 Department of Epidemiology, School of Public Health, Jiangsu Key Laboratory of Preventive and Translational Medicine for Geriatric Diseases, MOE Key Laboratory of Geriatric Diseases and Immunology, Suzhou Medical College of Soochow University, Suzhou, China; 5 School of Population Medicine and Public Health, Chinese Academy of Medical Sciences and Peking Union Medical College, Beijing, China

**Keywords:** Hearing loss, Diabetes, Diabetes duration, China National Health Survey

## Abstract

**Purpose:**

To examine the effect of diabetes, duration of diabetes, and blood glucose on speech-, low/mid-, and high-frequency hearing loss.

**Methods:**

In this cross-sectional study, 2821 participants aged 20–87 years in the China National Health Survey were included. Diabetes was defined as valid fasting blood glucose (FBG) of ≥ 7.0 mmol/L, a self-reported history of diabetes or the use of anti-diabetic medications. Speech-(500, 1000, 2000, and 4000 Hz), low/mid- (500, 1000 and 2000 Hz), and high-frequency (4000, 6000, and 8000 Hz) hearing loss was defined as pure tone average of responding frequencies > 20 dB HL in the better ear, respectively.

**Results:**

In fully adjusted models, for speech-, low/mid-, and high-frequency hearing loss, compared with no diabetes, those with diabetes (OR[95%CI]: 1.44 [1.12, 1.86], 1.23 [0.94, 1.61], and 1.75 [1.28, 2.41], respectively) and with diabetes for > 5 years duration (OR[95%CI]: 1.63 [1.09, 2.42], and 1.63 [1.12, 2.36], 2.15 [1.25, 3.70], respectively) were at higher risk. High FBG level was associated with a higher risk of speech-, low/ mid-, and high-frequency hearing loss. And there were stronger associations between HL and diabetes, longer duration and higher in “healthier population” (no hypertension, no dyslipidemia and younger age).

**Conclusion:**

Diabetes, longer duration, and higher FBG level were independently associated with hearing loss for speech-, low/mid- and high-frequency hearing loss, particularly in higher frequency and “healthier population”. Paying more attention to hearing loss in those populations could lower the burden of hearing loss.

**Supplementary Information:**

The online version contains supplementary material available at 10.1007/s40618-024-02406-2.

## Introduction

Hearing loss (HL) affects approximately 1.57 billion people globally, is the third cause of years lived with disability (YLDs), and can have serious consequences for health conditions (e.g., poorer physical function and social isolation) [1–3] and great social and economic impact [4]. Specifically, a recent Lancet report identified hearing loss as the largest modifiable risk factor for dementia [5]. There are many modifiable risk factors associated with HL, such as diabetes and other cardiometabolic risks. If we can identify the relationships between these risks and HL, it’s possible to prevent the incidence of HL. It’s estimated at 537 million adults with diabetes, and the prevalence of diabetes increasing with age [6]. The number will rise to 643 million by 2030 and 783 million by 2045 [6]. Remarkably, diabetic patients have twice the incidence of HL compared to those without diabetes [7].

The effects of diabetes on hearing loss have been acknowledged for decades, particularly at high-frequency HL [8]. The potential reasonable biological mechanisms of diabetes-related hearing loss are mainly microvascular etiology and neurological etiology. The etiology changes have negative effects on mitochondrial function and gene expression along with inflammation and oxidative stress [9, 10], which affects inner ear structure and function, and further leads to hearing loss. However, the association between diabetes and hearing loss remains controversial due to inconsistent measures and definitions of primary variables [11–13]. For example, participants were categorized by self-reported HL. Although there are inconsistencies in the study methods and definitions used, most relevant studies showed some association between hearing loss and diabetes although there are few exceptions [14]. And there were few studies (mainly about speech frequencies) to investigate the association between the duration of diabetes and HL, which was inconsistent [12, 15]. There was a lack of studies on associations of low/mid-frequency and high-frequency HL with duration.

Understanding the impact of diabetes, diabetes duration and blood glucose level could provide tools to prevent HL. There is limited evidence on the association between diabetes duration and HL, mainly at speech-frequency HL. Therefore, we examined the effect of diabetes, duration of diabetes, and blood glucose on speech-, low/ mid-, and high-frequency HL, respectively, using data from a community-based survey.

## Materials and methods

### Study design and participants

China National Health Survey (CNHS) is a large nationwide multi-ethnic population-based cross-sectional study, which used a multistage stratified cluster sample designed to be representative of the civilian China population [16]. This study data comes from the CNHS collected in Jiangsu province in 2023. Individuals from the selected communities and villages were all invited to participate. The inclusion criteria were adults aged ≥ 20 and those having lived in the local area for at least 5 years. The exclusion criteria were people with severe mental or physical disorders, pregnant women, persons on active military duty, or foreigners. Patients with previous ear diseases like otitis media, conductive HL, and ear surgeries were excluded additionally for our analysis. One-half of the about 6000 study participants were randomly assigned to audiometric testing. Ethical approval was obtained from the Bioethical Committee of the Institute of Basic Medical Sciences, Chinese Academy of Medical Sciences (No. 2022134). All study participants provided their written informed consent.

### Assessment of audiometry

Pure-tone hearing detection thresholds were determined using clinical diagnostic audiometers (Interacoustics, AD226, Denmark) by trained professional staff in sound-isolating rooms in which ambient noise did not exceed 40 dBA as measured by a sound level meter. Hearing aids were not worn during audiometric testing. Biological calibration listening checks were performed daily on the machines. Pure tone audiometry was measured for the audiometric threshold of each ear at 500, 1000, 2000, 4000, 6000, and 8000 Hz using a modified Hughson-Westlake method [17]. Each frequency contained an intensity range of −10 to 120 dB HL. If the participant did not respond at the maximum value, the audiometric threshold would be recorded as the maximum value for the purpose of our analyses. Individuals would be not tested if otitis media and/or cerumen are detected during the electric otoscopy.

We calculated 3 pure tone averages (PTA) according to different frequency ranges, with the first using the low/mid-frequency (500, 1000, and 2000 Hz) pure-tone average, the second considering the speech-frequency (500, 1000, 2000, and 4000 Hz) pure-tone average, and the third considering the high-frequency pure-tone average (4000, 6000, and 8000 Hz). We defined speech-, low/ mid-, and high-frequency HL in relation to cutoff points according to the World Health Organization [18]: the PTA greater than 20 dB HL reflecting HL. Participants were represented by the lower threshold or PTA of 2 ears.

### Assessment of diabetes

Participants were identified as having diabetes if they met any of the following criteria: (1) a valid fasting blood glucose (FBG) of ≥ 7.0 mmol/L; or (2) self-reported physician diagnosis; or (3) a positive response to the question, “Have you taken medication for diabetes?”. Gestational diabetes was not included. Fasting blood samples were collected from participants for measurements of FBG after at least 8 h fasting overnight.

The diabetes duration was calculated by subtracting the date of diagnosis from the date of investigation. The duration of new cases was calculated as 0 year. The duration of diabetes was categorized into no diabetes, 0 to 5 years, and > 5 years.

### Other variables

The investigation contained the questionnaire and the equipment was uniform and the criteria were consistent for the measurements that were used. Information on sociodemographic characteristics, lifestyle factors, and medical history was obtained from a standard questionnaire through face-to-face interviews. To ensure data accuracy, questionnaires were checked and verified by inspectors after the completion of each questionnaire. Participants reported their age, sex, educational level, smoking status, alcohol use, tea, coffee, frequency of earphone use, exposure to occupational noise, presence of tinnitus and exposure to ototoxic medication (mainly about aminoglycoside antibiotics, including amikacin, neomycin, kanamycin, gentamicin and streptomycin, and platinum-based anticancer drugs, including cisplatin and carboplatin) by a standard health questionnaire. Outer and middle ear function, which was assessed by electric otoscopy, was categorized into “normal” or “abnormal” after excluding those with otitis media and cerumen. Menopause status was assessed among women with the question: “Have your menstrual periods stopped for at least 1 year and did not restart?” with response options “Yes,” or “No”. Missing values were regarded as menopause (i.e., “Yes”) if the age was above 55 years since 90% of Chinese women experience menopause during 45–55 years [19]. Weight and height were measured under standardized conditions, and the body mass index (BMI) was calculated as weight (kg) divided by height (m) squared. Participants were identified as having hypertension if they met any of the following criteria: (1) an average systolic blood pressure (SBP) ≥ 140 mm Hg and/or an average diastolic blood pressure (DBP) ≥ 90 mm Hg; or (2) self-reported physician diagnosis; or (3) a positive response to the question, “has you taken medication for hypertension?”. DBP and SBP were measured 3 times at each survey community or village by trained technicians who used an automated electronic device (OMRON, HEM-907). Average diastolic and systolic blood pressure were calculated from the final valid three measurements. Participants were identified as having dyslipidemia if they met any of the following criteria: (1) a positive response to the question, “Has a doctor ever told you that you have dyslipidemia?”; or (2) a positive response to the question, “have you taken medication for dyslipidemia?”.

### Statistical analysis

2956 participants completed the audiometric examination and the structured questionnaire and are included in this analysis. Of them, we excluded 135 participants with a history of any signal of ear diseases, such as sudden HL and otitis media. This resulted in an analytical sample of 2821 men and women. Among those participants, missing values of potential covariate, BMI(*n* = 4[0.14%]) were imputed as the reference group to be conservative [20], in other words, the missing values of BMI were imputed as the normal group, 18.5–23.9 kg/m^2^.

Participants were classified according to characteristics of 3 PTAs as normal and HL. Differences in sociodemographic characteristics, lifestyle and prevalence of different diseases across categories of 3 PTAs were reported as a mean and standard deviation for continuous variables and percentage for categorical variables. We used the Chi-square test to calculate the *P* values for categorical and the Student’s *T* test or Wilcoxon rank-sum test for continuous variables, as appropriate.

We estimated odds ratios (ORs) and their 95% confidence interval (CI) for the association between diabetes and duration and HL by logistic regression models. Three models were built respectively to adjust for potential confounders. The first model was adjusted for age (< 65/ ≥ 65 years) and sex. The second model was additionally adjusted for education level, smoking (current, former, never), alcohol consumption (current, former, never), tea (current, former, never), coffee (no/yes), BMI (≤ 18.5, 18.5 to < 24, ≥ 24 kg/m^2^), use of earphone (no/yes), tinnitus (no/yes), electric otoscopy (normal/abnormal), occupational noise exposure (no/yes) and use of ototoxic medication (no/yes). The third model was further adjusted for hypertension (no/yes) and dyslipidemia (no/yes). We also modeled FBG as a continuous variable to calculate the risk of HL associated with a 1 mmol/L increment for the FBG.

We conducted the analyses separately in age groups and sex. The interaction effects of diabetes-related issues (diabetes, duration, and FBG) and age group, sex, and other cardiometabolic risks (hypertension, BMI, and dyslipidemia) on HL were also tested. We further carried out stratified analyses by hypertension group or dyslipidemia group in the low/mid-frequency and hypertension group or sex in the high-frequency, since we found statistically significant interaction effect of these corresponding items on HL (Table S1).

We also conducted several sensitivity analyses: (a) excluding all participants with abnormal ear examination or missing values, to understand whether the effect of diabetes and FBG level depend on the ear examination; (b) removing from the models the adjustment for tinnitus, since it is unclear if this disorder might be part of the HL process; (c) adjusting for menopausal status (no/yes) in the third model in women additionally, since menopause may affect hearing health.

All tests were two-sided, with *p* < 0.05 considered statistically significant. Analyses were performed with SAS (version 9.4; SAS Institute Inc., Cary, NC, United States).

## Results

Characteristics of the population aged 20–87 years in Jiangsu province, China are presented in Table 1, stratified by speech-, low/ mid-, and high-frequency HL. People with HL were, on average, 6–12 years older than those without HL, the min age difference is the category of high-frequency HL. 70.03% of adults without high-frequency HL were women, which was over three times the proportion of men. We found no difference in low/ mid-frequency HL by sex [21]. The median duration of participants with diabetes is 5 years and longer duration have a larger proportion of participants with HL compared to those without HL. The mean FBG level is 5.78 mmol/L. At high-frequency HL, there was the lowest FBG level (mean (SD): 5.98 (1.57)).

**Table 1:** Basic characteristics of the study population by speech-, low/mid- and high-frequency hearing status

Characteristic	Overall	Hearing status								
		Speech-frequency PTA			Low/mid-frequency PTA			High-frequency PTA		
		Normal	Hearing loss	*P* value	Normal	Hearing loss	*P* value	Normal	Hearing loss	*P* value
No. of participants	2821	1539 (54.56)	1282 (45.44)		1707 (60.51)	1114 (39.49)		1031 (36.55)	1790 (63.45)	
Age, mean (SD), year	53.10 (12.77)	47.16 (12.08)	60.23 (9.52)	< 0.0001	48.54 (12.23)	60.09 (10.15)	< 0.0001	43.13 (11.36)	58.84 (9.62)	< 0.0001
Age group, No. (%)				< 0.0001			< 0.0001			< 0.0001
< 65 years	2268 (80.40)	1432 (93.05)	836 (65.21)		1556 (91.15)	712 (63.91)		1004 (97.38)	1264 (70.61)	
≥ 65 years	553 (19.60)	107 (6.95)	446 (34.79)		151 (8.85)	402 (36.09)		27 (2.62)	526 (29.39)	
Women, No. (%)	1641 (58.17)	1014 (65.89)	627 (48.91)	< 0.0001	1016 (59.52)	625 (56.10)	0.0722	722 (70.03)	919 (51.34)	< 0.0001
High school or above, No. (%)	1306 (46.30)	868 (56.40)	438 (34.17)	< 0.0001	917 (53.72)	389 (34.92)	< 0.0001	661 (64.11)	645 (36.03)	< 0.0001
Smoking status, No. (%)				< 0.0001			< 0.0001			< 0.0001
Never	2193 (77.74)	1302 (84.60)	891 (69.50)		1365 (79.96)	828 (74.33)		894 (86.71)	1299 (72.57)	
Former	216 (7.66)	64 (4.16)	152 (11.86)		98 (5.74)	118 (10.59)		31 (3.01)	185 (10.34)	
Current	412 (14.60)	173 (11.24)	239 (18.64)		244 (14.29)	168 (15.08)		106 (10.28)	306 (17.09)	
Alcohol intake, No. (%)				< 0.0001			< 0.0001			< 0.0001
Never	1963 (69.59)	1157 (75.18)	806 (62.87)		1223 (71.65)	740 (66.43)		808 (78.37)	1155 (65.53)	
Former	136 (4.82)	38 (2.47)	98 (7.64)		51 (2.99)	85 (7.63)		21 (2.04)	115 (6.42)	
Current	722 (25.59)	344 (22.35)	378 (29.49)		433 (25.37)	289 (25.94)		202 (19.59)	520 (29.05)	
Tea, No. (%)				0.0059			0.0019			0.0129
Never	1988 (70.47)	1077 (69.98)	911 (71.06)		1183 (69.30)	805 (72.26)		721 (69.93)	1267 (70.78)	
Former	50 (1.77)	17 (1.10)	33 (2.57)		21 (1.23)	29 (2.60)		9 (0.87)	41 (2.29)	
Current	783 (27.76)	445 (28.91)	338 (26.37)		503 (29.47)	280 (25.13)		301 (29.19)	482 (26.93)	
Coffee, No. (%)	552 (19.57)	404 (26.25)	148 (11.54)	< 0.0001	404 (23.67)	148 (13.29)	< 0.0001	332 (32.20)	220 (12.29)	< 0.0001
BMI, kg/m^2^				< 0.0001			< 0.0001			< 0.0001
< 18.5	73 (2.59)	50 (3.25)	23 (1.79)		53 (3.10)	20 (1.80)		39 (3.78)	34 (1.90)	
18.5 ~ 23.9	1169 (41.44)	714 (46.39)	455 (35.49)		777 (45.52)	392 (35.19)		499 (48.40)	670 (37.43)	
≥ 24	1579 (55.97)	775 (50.32)	804 (62.71)		877 (51.38)	702 (63.02)		493 (47.82)	1086 (60.67)	
Occupational noise exposure, No. (%)	244 (8.65)	74 (4.81)	170 (13.26)	< 0.0001	121 (7.09)	123 (11.04)	0.0003	25 (2.42)	219 (12.23)	< .0001
Tinnitus, No. (%)	304 (10.78)	103 (6.69)	201 (15.68)	< 0.0001	132 (7.73)	172 (15.44)	< 0.0001	56 (5.43)	248 (13.85)	< 0.0001
Abnormal electric otoscopy, No. (%)	24 (0.85)	12 (0.78)	12 (0.94)	0.6526	15 (0.88)	9 (0.81)	0.8413	9 (0.87)	15 (0.84)	0.9225
Use of ototoxic drug, No. (%)	520 (18.43)	226 (14.68)	294 (22.93)	< 0.0001	266 (15.58)	254 (22.80)	< 0.0001	122 (11.83)	398 (22.23)	< 0.0001
Use of earphone, No. (%)	355 (12.58)	261 (16.96)	94 (7.33)	< 0.0001	272 (15.93)	83 (7.45)	< 0.0001	209 (20.27)	146 (8.16)	< 0.0001
Hypertension, No. (%)	1236 (43.81)	514 (33.40)	722 (56.32)	< 0.0001	623 (36.50)	613 (55.03)	< 0.0001	270 (26.19)	966 (53.97)	< 0.0001
Dyslipidemia, No. (%)	605 (21.45)	260 (16.89)	345 (26.91)	< 0.0001	289 (16.93)	316 (28.37)	< 0.0001	141 (13.68)	464 (25.92)	< 0.0001
Duration, media (IQR), years	5 (8)	4 (9)	6 (9)	0.0069	5 (6.5)	6 (10)	0.0011	4 (7)	6 (8)	0.0926
FBG, mean (SD), mmol/L	5.78 (1.44)	5.54 (1.15)	6.06 (1.67)	< 0.0001	5.61 (1.24)	6.02 (1.66)	< 0.0001	5.43 (1.08)	5.98 (1.57)	< 0.0001

BMI indicates body mass index; FBG indicates fasting blood glucose

**Fig. 1:**
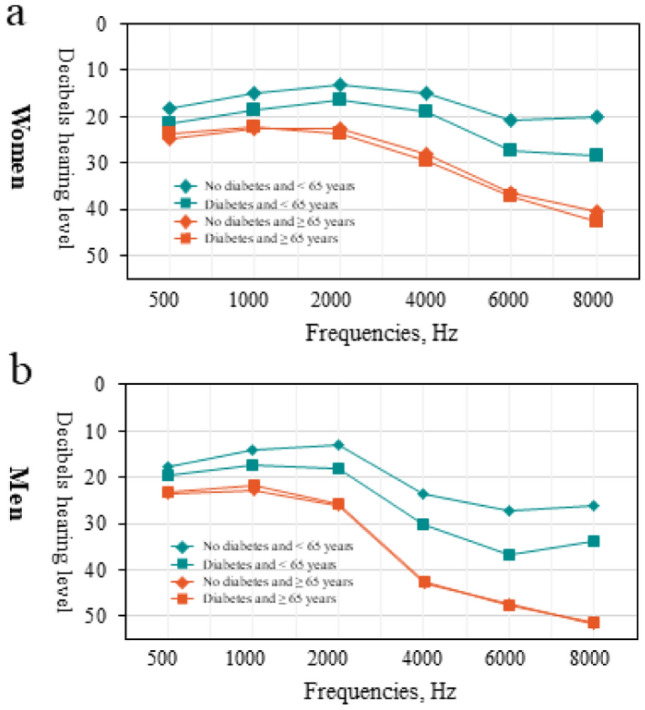
Average audiograms of octave frequencies from 500 to 8000 Hz for better hearing ears by age group (< 65/ ≥ 65 years) and sex. The parameter in each panel is presence of diabetes or not

There were 401 participants with diabetes, 236 participants with diabetes for 0 to 5 years and 156 participants with diabetes for > 5 years duration (Table 2). In general, we found there was a higher hearing threshold in those participants who had diabetes compared with those without diabetes no matter sex sex-specific or age-specific it is, and that the magnitude of the associations between diabetes and HL is the strongest compared with those of other cardiometabolic diseases and HL (Fig. 1 and Table S7). In fully adjusted models, compared with those who did not have diabetes, those with diabetes were at higher risk for HL, the multivariable-adjusted ORs (95% CI) were: 1.44 (1.12, 1.86) and 1.75 (1.28, 2.41) in speech- and high-frequency HL respectively, but in low/mid-frequency HL (OR[95% CI]: 1.23 [0.94, 1.61]) (Table 2). There was a higher risk of HL in those participants who had diabetes for > 5 years duration compared with those without diabetes, the multivariable-adjusted OR were 1.63 (1.09, 2.42), 1.63 (1.12, 2.36), 2.15 (1.25, 3.70) in speech-, low/mid-, and high-frequency HL, respectively (Table 2). By contrast, there was no significantly higher risk in those participants who had diabetes for 5 or less years compared with those without diabetes except for high-frequency HL (OR (95% CI): 1.58 (1.08, 2.33)). We estimated that 1 mmol/L increment in FBG level was associated with a higher risk of speech-, low/ mid-, and high-frequency HL, the multivariable-adjusted ORs (95% CI) were: 1.12 (1.05, 1.20), 1.10 (1.03, 1.16) and 1.14 (1.05, 1.24), respectively (Table 2 and Fig. 3).

**Table 2:** Associations between diabetes, duration and FBG and speech-, low/mid- and high-frequency hearing loss

Variable	No diabetes	Diabetes		Duration group				Continuous per 1-mmol/L increment	
				0 to 5 years duration		> 5 years duration			
		OR (95% CI)	*P* value	OR (95% CI)	*P* value	OR (95% CI)	*P* value	OR (95% CI)	*P* value
Participants, n/N	2420/2821	401/2821		236/392		156/392		2820/2820	
Speech frequency hearing loss									
Model 1	1 (ref)	**1.84 (1.45, 2.33)**	< 0.0001	**1.83 (1.37, 2.47)**	< 0.0001	**1.93 (1.32, 2.83)**	0.0007	**1.22 (1.15, 1.30)**	< .0001
Model 2	1 (ref)	**1.57 (1.23, 2.02)**	0.0004	**1.48 (1.09, 2.02)**	0.0127	**1.78 (1.20, 2.65)**	0.0041	**1.15 (1.08, 1.23)**	< .0001
Model 3	1 (ref)	**1.44 (1.12, 1.86)**	0.0046	**1.37 (1.00, 1.86)**	0.0494	**1.63 (1.09, 2.42)**	0.0166	**1.12 (1.05, 1.20)**	0.0005
Low/mid-frequency hearing loss									
Model 1	1 (ref)	**1.59 (1.26, 2.00)**	< 0.0001	**1.44 (1.08, 1.92)**	0.0135	**1.92 (1.34, 2.76)**	0.0004	**1.17 (1.10, 1.24)**	< .0001
Model 2	1 (ref)	**1.35 (1.04, 1.76)**	0.0245	1.20 (0.89, 1.62)	0.2245	**1.79 (1.24, 2.60)**	0.0020	**1.12 (1.05, 1.18)**	0.0002
Model 3	1 (ref)	1.23 (0.94, 1.61)	0.1258	1.11 (0.83, 1.50)	0.4797	**1.63 (1.12, 2.36)**	0.0110	**1.10 (1.03, 1.16)**	0.0024
High-frequency hearing loss									
Model 1	1 (ref)	**2.55 (1.90, 3.42)**	< 0.0001	**2.44 (1.70, 3.48)**	< 0.0001	**3.05 (1.82, 5.10)**	< .0001	**1.34 (1.23, 1.47)**	< .0001
Model 2	1 (ref)	**2.06 (1.51, 2.82)**	< 0.0001	**1.85 (1.26, 2.70)**	0.0016	**2.59 (1.52, 4.41)**	0.0005	**1.20 (1.11, 1.31)**	< .0001
Model 3	1 (ref)	**1.75 (1.28, 2.41)**	0.0005	**1.58 (1.08, 2.33)**	0.0056	**2.15 (1.25, 3.70)**	0.0056	**1.14 (1.05, 1.24)**	0.0021

*P* < 0.05 was considered statistically significant, where has been bolded

OR (95% CI) were from logistic regression models

Multivariable (MV) adjusted model 1: adjusted for age (< 65/ ≥ 65 years) and sex

MV adjusted model 2: as model 1, additionally adjusted for education level, smoking (current, former, never), alcohol consumption (current, former, never), tea (current, former, never), coffee (no/yes), BMI (≤ 18.5, 18.5 to < 24,  ≥ 24 kg/m^2^), use of earphone (no/yes), tinnitus (no/yes), electric otoscopy (normal/abnormal), occupational noise exposure (no/yes) and use of ototoxic medication (no/yes)

MV adjusted model 3: as model 2, further adjusted for hypertension (no/yes) and dyslipidemia (no/yes)

The number of participants in models is 2821 (2420 + 401) when comparison is between diabetes and no diabetes, 2812 (2420 + 236 + 156) when comparison is between duration groups and no diabetes, 2820 when modeled FBG as a continuous variable. The inconsistency of the number of participants across different models is due to the missing of explanatory variable values

*BMI* indicates body mass index, *FBG* indicates fasting blood glucose, *OR* indicates odds ratios, *CI* indicates confidence intervals

The stratified analysis parameters were selected according to the results of multivariate analysis. About age-specific risk, a significantly stronger positive association between diabetes (diabetes vs no diabetes) and risk of HL was observed in participants aged < 65 years at speech-, low/mid- and high-frequency HL (OR (95% CI): 1.50 (1.12, 2.00), 1.45 (1.09, 1.93), 1.82 (1.30, 2.55), respectively). There was a higher risk of HL in those participants who had diabetes for > 5 years duration compared with those without diabetes, the multivariable-adjusted OR were 1.70 (1.06, 2.73), 1.88 (1.18, 3.00) and 2.33 (1.29, 4.22) at speech-, low/mid-, and high-frequency HL, respectively (Table S2). By contrast, we did not observe a significant association in participants aged ≥ 65 years. Regarding sex-specific risk, in the women population, we observed a significantly positive association between diabetes (diabetes vs no diabetes) and risk of HL (OR (95% CI): 1.43 (1.01, 2.05)) and 1 mmol/L increment in FBG level was associated with higher risk (OR (95% CI): 1.11 (1.01, 1.23)) at speech-frequency HL. In men population, there was a higher risk of HL in those participants who had diabetes, diabetes for 5 or less years and diabetes for > 5 years duration compared with those without diabetes at high-frequency, the multivariable-adjusted OR (95% CI) was 2.55 (1.51, 4.30), 2.28 (1.20, 4.33) and 3.39 (1.40, 8.19), respectively. And we also found that 1 mmol/L increment in FBG level was associated with higher risk (OR (95% CI): 1.12 (1.03, 1.22), 1.11 (1.03, 1.20) and 1.22 (1.06, 1.40), respectively) at speech-, low/mid-, and high-frequency HL (Table S3).

**Fig. 2:**
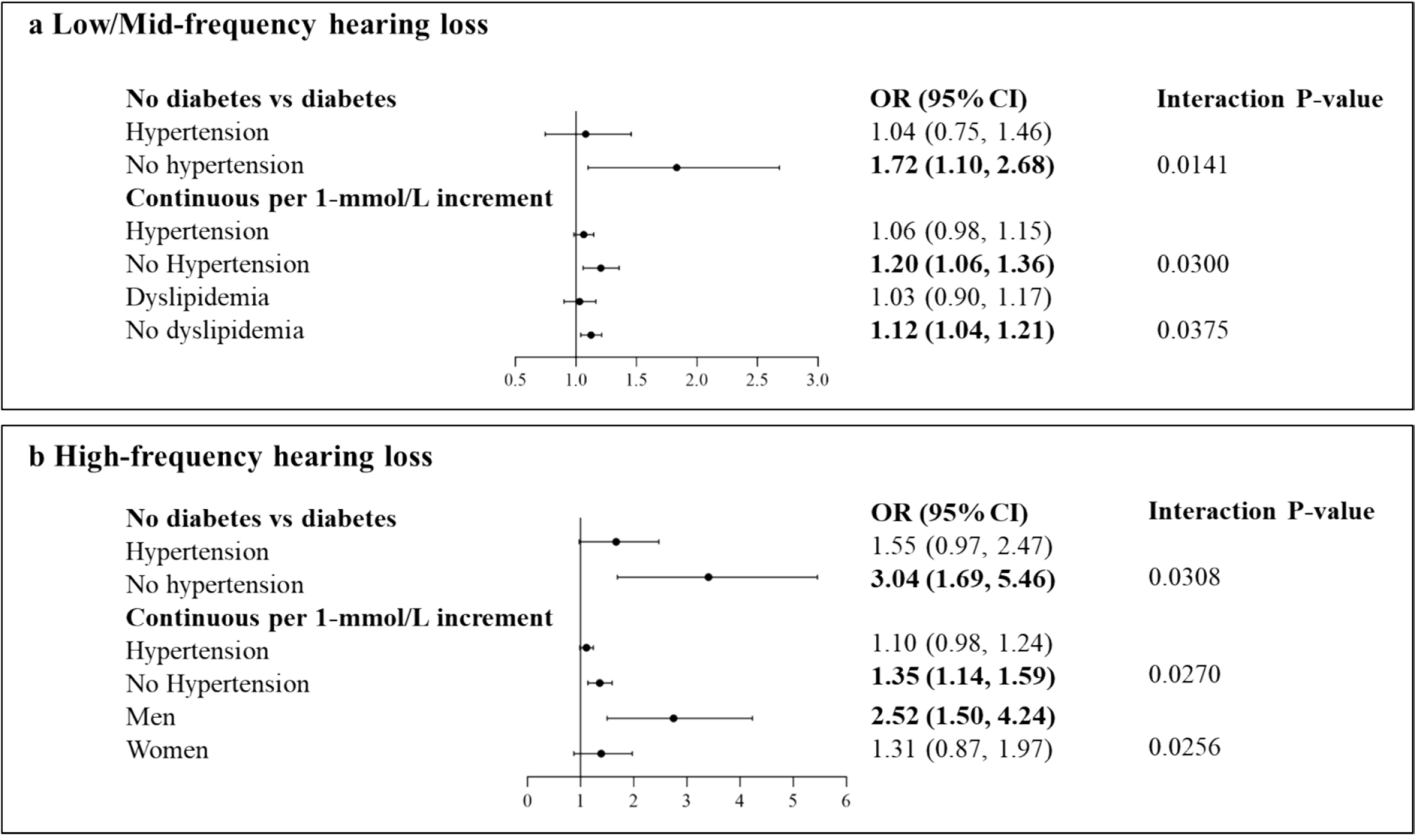
Odds ratios (95% confidence interval) for the association between interact terms in low/mid- and high-frequency hearing loss and risk of hearing loss. Logistic regression models adjusted for all related covariates except corresponding interact terms

Additionally, we found statistically significant interactions between the hypertension group or dyslipidemia group or sex and diabetes group or continuous FBG level to HL (Fig. 2). At low/mid-frequency HL, a significantly stronger positive association between diabetes (diabetes vs no diabetes) and risk of HL (OR (95% CI): 1.72 (1.10, 2.68)) was observed in participants without hypertension, and 1 mmol/L increment in FBG level was associated with higher risk in participants without hypertension and participants without dyslipidemia, the multivariable-adjusted ORs (95% CI) were: 1.20 (1.059, 1.358) and 1.12 (1.04, 1.21), respectively. At high-frequency HL, compared with those who did not have diabetes, those with diabetes were at higher risk for HL, the multivariable-adjusted ORs (95% CI) were: 3.03 (1.69, 5.46)) in participants without hypertension and 1 mmol/L increment in FBG level was associated with higher risk in participants without hypertension and men, the multivariable-adjusted ORs (95% CI) were: 1.35 (1.14, 1.59) and 2.52 (1.50, 4.24), respectively.

These results were similar when we only included the analysis participants with normal ear examination (Table S4). Removing from the models the adjustment for tinnitus did not modify the estimates of the studied association (Table S5). Further, adjusting for menopausal status (no/yes) in the third model in women did not modify the effect size of the studied association almost (Table S6).

## Discussion

**Fig. 3:**
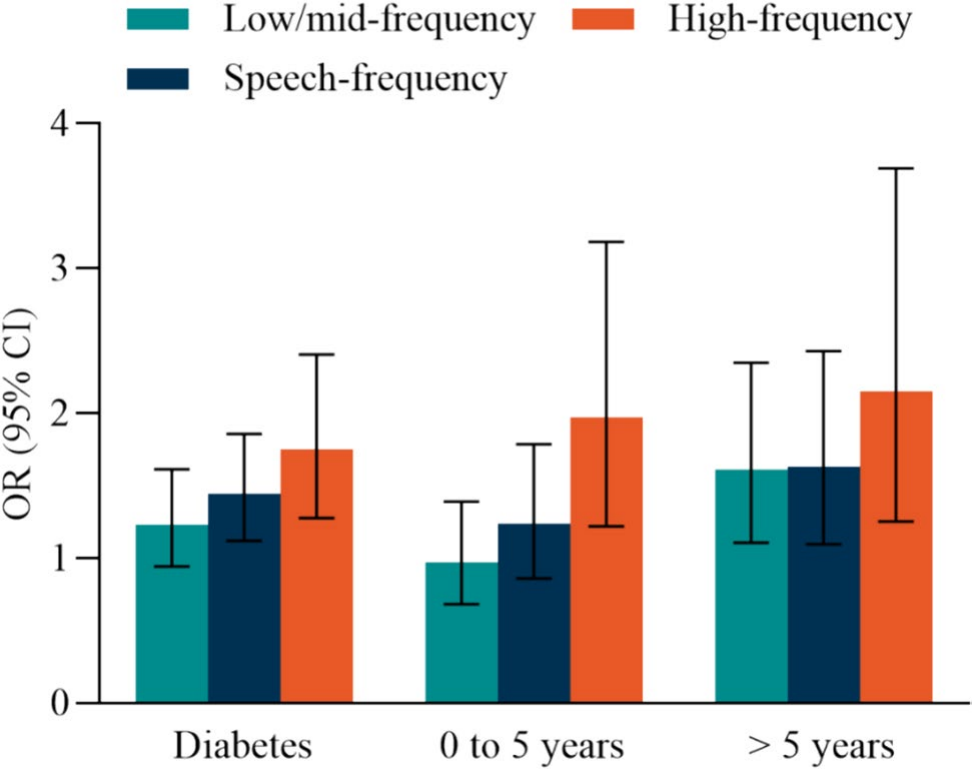
The associations of diabetes and duration with speech-, low/mid- and high-frequency hearing loss. OR (95% CI) were from logistic regression models, which were adjusted for age(< 65/ ≥ 65 years), education level, smoking (current, former, never), alcohol consumption (current, former, never), tea (current, former, never), coffee (no/yes), BMI (≤ 18.5, 18.5 to < 24, ≥ 24 kg/m^2^), use of earphone (no/yes), tinnitus (no/yes), electric otoscopy (normal/abnormal), occupational noise exposure (no/yes), use of ototoxic medication (no/yes), hypertension (no/yes) and dyslipidemia (no/yes). OR indicates odds ratios; CI indicates confidence intervals

To our knowledge, this is the first study to examine the impact of diabetes on hearing using the criteria of the World Report on hearing [18]. In summary, there was a higher hearing threshold in those participants who had diabetes no matter sex sex-specific or age-specific it is. We found that diabetes, longer duration and higher FBG were independently associated with hearing loss for speech-frequency and high-frequency HL, and the associations were stronger at high-frequency HL (Fig. 3). At low/mid-frequency HL, only > 5 years duration of duration and higher FBG were associated with HL independently. Furthermore, the “healthier population” (no hypertension, no dyslipidemia and younger age) has stronger associations and higher risks.

The potential reasonable biological mechanisms of diabetes-related hearing loss are mainly clustered to two aspects, neurological etiology and microvascular etiology. Diabetic neuropathy is mainly caused by diabetes-related metabolism imbalance and an imbalance in the mitochondrial redox state [9]. These imbalances collectively culminate in negative effects on mitochondrial function and gene expression along with inflammation and oxidative stress [9, 10], further leading to hearing loss. Atrophy of the spiral ganglion and demyelination of the eighth cranial nerve among autopsied diabetic patients suggest a neurological etiology to diabetes-related hearing impairment [22]. Microvascular changes in the cochlea occur at the early stage of diabetes, and studies have found thickened basilar membranes and capillaries of the stria vascularis and atherosclerotic narrowing of the internal auditory artery among autopsied people who had diabetes, but not in people without diabetes [22]. These microvascular changes reduce blood supply in the cochlea and further lead to hypoxia and insufficient energy supply, which in turn affects inner ear function. These negative changes involving abnormality of metabolism, blood supply and the cochlea structure would lead to hearing loss gradually. More terribly, mitochondrial dysfunction facilitates further mitochondrial ROS production in a positive feedback loop and affects metabolism and energy supply, ultimately resulting in the activation of cells' apoptotic pathways in cochlear and subsequent hearing loss, namely a vicious circle.

In our study, the effect size (OR) was small relatively, which may result from the difference in age range, inconsistent definitions of HL, different categorized criteria of speech-, low/mid- and high- frequency and unadjusted important potential confounders (e.g., BMI, ototoxic drug and hypertension) among studies. Nonetheless, the results were consistent with previous studies overall. A meta-analysis, in which data were obtained from cross-sectional studies, suggested a higher risk of HL in those participants with diabetes compared with those without diabetes (OR (95%CI): 2.15 (1.72–2.68)) [23]. Dalton et.al also found there was an association between NIDDM and hearing loss when adjusting for potential confounders (OR(95% CI): 1.41 (1.05–1.88))[15] at speech-frequency HL, although they did not finely adjust for BMI and ototoxic drugs. The evidence from National Health and Nutrition Examination Surveys (NHANES), which enrolled younger participants than ours, showed OR (95%CI) of 1.82 (1.27, 2.60) and 2.16 (1.47, 3.18) without adjusting chronic diseases (e.g., hypertension and dyslipidemia) for the low/mid-frequency (500, 1000, 2000 Hz) and high frequency (3000, 4000, 6000, 8000 Hz) HL(PTA > 25 dB HL), respectively [8]. In addition, we also found that the magnitude of the associations between diabetes and HL is the strongest compared with those of other cardiometabolic diseases and HL, which suggested that the associations of diabetes and hearing loss are really disease-dependent and not a reflex of the aging process itself.

The impact of diabetes duration on HL was inconsistent in present studies may be due to inconsistency of method and population. The results from NHS I and II [12] found that a longer duration (for ≥ 8 years) of diabetes was associated with a higher risk of moderate self-reported HL. Mitchell et.al. also found an association between diabetes and hearing loss for diabetes duration ≥ 10 years (OR (95% CI): 2.08 (1.10–3.94)) at speech-frequency HL (PTA > 25 dB HL) [24]. It was observed that the degree of high-frequency HL (> 2000 Hz) was related to the duration(< 1 year, 1–5 years, 6–10 years and > 10 years) [25] proportionally in a clinical study. Likewise, HAl-Sofiani et.al found that high-frequency hearing loss was significantly and positively correlated with age and duration of type 1 diabetes [26]. By contrast, Dalton et.al did not find associations between the duration of diabetes (1-year change) and hearing loss (OR (95% CI) 0.99 (0.96–1.02)) [15] at speech-frequency HL, which could result from designed short term of duration group. Also, the cross-sectional study from Brazil showed there were no significant differences in mean hearing threshold (duration ≥ 9 years vs duration < 9 years) after adjusting for age, gender, and hypertension at octave frequencies from 250 to 8000 Hz, although the mean hearing threshold was higher in duration ≥ 9 years group [27]. In our study, we found longer diabetes duration impaired hearing, which has a mild dose–response relationship. We have no idea to identify the reason for the inconsistent observations and more studies are needed to explore the relationship. However, the observed associations suggested diabetes-mediated changes in hearing occurred over the long term, namely negative cumulative effect.

A higher FGB level is associated with a higher risk of HL in all frequencies, which is similar to previous studies. The fifth Korea National Health and Nutrition Examination Survey, where HL was defined as the average hearing threshold at 500, 1000, 2000, 3000, 4000 and 6000 Hz exceeding 25 dB HL, suggested increased fasting glucose was independently associated with HL (OR (95% CI): 1.4 (1.1–1.8)) in participants with metabolic components [28]. Similarly, data from the Dongfeng‑Tongji cohort study in China found significant dose-dependent relationships between increasing high-frequency ((4000 and 8000 Hz) hearing loss levels [29]. Cochlea metabolic disturbance and the death of hair cells induced by long-term exposure to pathoglycemia may lead to HL.

Several specific findings were also observed. At low/mid-frequency HL, there was no association between diabetes and HL while a higher risk in those with diabetes for > 5 years duration. Also, there was a significant association between diabetes duration ≤ 5 years and HL compared with those without diabetes at high-frequency HL only. In addition, the impacts of diabetes-related risks on hearing were strongest at high-frequency HL. Many epidemiology studies have shown higher frequency threshold differences between diabetes and those without diabetes were larger than lower frequency [8, 24, 30, 31]. It suggested that the main frequency affected by diabetes will gradually progress from high frequency to low frequency with onset. In other words, the first damaged structures in the inner ear are those located at the cochlear basal turn, which is responsible for the high-frequency threshold. The mitochondrial damage increased with time and the damage in the cochlea basal turn was the most severe [32]. The guinea pig model has shown 5 h-room temperature survival rate of OHCs from the base was less than one-third of that from the apex, which suggested that basal OHCs may be more vulnerable to free-radical damage than apical OHCs [33]. Also, outer hair cells (OHCs) play a crucial role in cochlear amplification, which is responsible for the exquisite sensitivity and frequency selectivity.

The stronger associations between HL and diabetes, longer duration and higher FBG in those “healthier populations” (no hypertension, no dyslipidemia and younger age) are, to our knowledge, uncommon findings, while the associations were stronger in men rather than in women may due to the protective effect (otoprotection and neuroprotection) of estrogen on hearing function [34]. There are several studies shown similar results. The study from America, which investigated the association between hearing loss and CVD comorbidities in 80 years and above older individuals, showed the effect value became no significance obviously in the multiple linear regression model for both high- and low-frequency pure-tone average (β (95% CI): 3.24 (− 5.84 to 12.33) and 3.69 (− 4.82 to 12.20)) [35]. Similarly, the study from the Canadian Longitudinal Study on Aging (CLSA) found stronger associations between PTA and poor health status in the younger age group rather than the older age group in the female [36]. There are several plausible interpretations underlying with these findings. It’s not consistent between chronological age and biological age which may result from poor health effects and age acceleration [37]. Thus, adjusting for chronological age is not completely equal to adjusting for biological age of hearing function. Diabetes has been associated with an increased risk of many negative outcomes, such as CVD [38], obesity, etc., which also affect the hearing effect. In older samples, the difference of the associations arising from other important causes of age-related HL (competing risk factors) may mask the contribution of diabetes to HL if other risks are imperfectly measured and thus not completely controlled in statistical models [31]. The potential competing effect was also implied by the stronger associations between diabetes and HL in those who without hypertension and dyslipidemia. Namely, in those who with other cardiometabolic diseases, the associations between diabetes-related risks and HL in participants with higher risks may be affected by competing risk factors with more probability. And it suggested that we should pay more attention to hearing function in the younger age with diabetes so that we can make earlier findings, perform earlier treatment and lower the burden of HL. We also found one more interact item exists when calculating the risk of HL associated with a continuous variable, FBG, at low/mid- and high-frequency HL. It may be because categorized variables may lose the original information [39] and lead to one of the categories sample small although it makes analysis and interpretation easier and mimics medical practice, and continuous variables use the information more sufficiently, and have more statistical power [39, 40].

Our study has several strengths. First, we examined octave frequencies from 500 to 8000 Hz, from low frequency to high frequency, so that we can explore the association between diabetes-related risk factors and different frequency HL collectively. Second, we categorized HL using the latest classification of HL in World Report on hearing [18]. In previous studies, the definition or classification of HL was not consistent, which weakens the conviction of the impact of diabetes on HL to an extent and leads to inconsistent conclusions and hard interpretations. Furthermore, we gave evidence to the relationship of diabetes duration with all frequency HL while the few related studies have different results.

There are also several limitations. First and most, FBG reflects levels at a single point in time and it does not indicate how glucose varied over time. So, the association between the hearing and FBG is likely stronger than we have found. The significant association between FBG and HL suggested that it is sufficiently accurate to be used. In further studies, we could measure FBG over time longitudinally or glycated hemoglobin to more precisely contrast the decline in hearing associated with diabetes-related risk factors, and to determine whether effective treatment of diabetes may delay the onset and progression of hearing loss. Secondly, we did not distinguish type 2 diabetes and type 2 diabetes. The potential mechanisms linked type 1 diabetes to HL and linked type 2 diabetes to HL may be different. In further studies, we should definite the type of diabetes for more clarity. Thirdly, our study was performed in Jiangsu province in China, and the findings may not apply to region or race/ethnicity groups. Studies in multiple regions and races are needed to examine the relationship between diabetes and HL and to explore the differences in varying populations. Finally, residual confounding cannot be excluded due to the observational design, although we have controlled important potential confounders (e.g., ototoxic drugs, exposure to occupational noise, etc.). And we have robust results.

## Conclusion

There was a higher hearing threshold in those participants who had diabetes no matter sex-specific or age-specific it is. Diabetes, longer duration and higher FBG were independently associated with hearing loss for speech-, low/mid- and high-frequency, and the impacts of diabetes-related risks on hearing were strongest at high-frequency HL. The stronger associations in those “healthier populations” suggested that the burden of disease is worse than we thought. We should pay more attention to hearing function among the participants with diabetes and longer duration than ever. Findings need to be corroborated using longitudinal studies. In future studies, we could use longitudinal design to explore the impact of different age onset of diabetes and to obtain the rank order of attribution of diabetes factors, to recognize the relationship clearer and prevent more precisely.

## Supplementary Information

Below is the link to the electronic supplementary material.

Supplementary file1 (DOCX 42 KB)

## Data Availability

The data that support the findings of this study are available on reasonable request from the corresponding author.
