# Supplementary material for: Diabetes and long duration leading to speech-, low/mid-, and high- frequency hearing loss: current evidence from the China National Health Survey 2023
Source: J Endocrinol Invest. 2024 Jun 13;48(1):233–43. doi: 10.1007/s40618-024-02406-2 (PMC11729146; doi:10.1007/s40618-024-02406-2)
Supplement: Supplementary file 1 — Supplementary file1 (DOCX 42 KB) [file 40618_2024_2406_MOESM1_ESM.docx]

**Supplementary materials**

**Article title** Diabetes and Long Duration Leading to Speech-, Low/Mid-, and High- frequency Hearing Loss: Current Evidence from the China National Health Survey 2023

**Journal name** Journal of Endocrinological Investigation

**Corresponding author** Xingming Chen

**Affiliations** Department of Otolaryngology-Head and Neck Surgery, State Key Laboratory of Complex Severe and Rare Diseases, Peking Union Medical College Hospital, Peking Union Medical College and Chinese Academy of Medical Sciences, Beijing, China.

**Email** [xingming.chen@hotmail.com](mailto:xingming.chen@hotmail.com)

**Corresponding author** Guangliang Shan

**Affiliations** Department of Epidemiology and Statistics, Institute of Basic Medical Sciences Chinese Academy of Medical Sciences, School of Basic Medicine Peking Union Medical College, Beijing, China

**Email** [guangliang_shan@163.com](mailto:guangliang_shan@163.com)

**Table S1 Interaction p-values between diabetes-related issues and age group, sex, and cardiometabolic risks**

| Variable | Diabetes | Duration group | FBG |
| --- | --- | --- | --- |
| Speech frequency hearing loss |  |  |  |
| Age group | 0.3445 | 0.6646 | 0.4810 |
| Sex | 0.9455 | 0.8733 | 0.6865 |
| Hypertension | 0.0961 | 0.5972 | 0.2758 |
| BMI | 0.4036 | 0.7633 | 0.6913 |
| Dyslipidemia | 0.4163 | 0.7199 | 0.2366 |
| Low/mid-frequency hearing loss |  |  |  |
| Age group | 0.2190 | 0.0709 | 0.1669 |
| Sex | 0.6550 | 0.9171 | 0.6413 |
| Hypertension | **0.0141** | 0.2569 | **0.0300** |
| BMI | 0.3804 | 0.7739 | 0.9575 |
| Dyslipidemia | 0.2478 | 0.1620 | **0.0375** |
| High-frequency hearing loss |  |  |  |
| Age group | 0.0969 | 0.5312 | 0.1406 |
| Sex | **0.0256** | 0.1358 | 0.1068 |
| Hypertension | **0.0308** | 0.0605 | **0.0270** |
| BMI | 0.5870 | 0.1013 | 0.6076 |
| Dyslipidemia | 0.5773 | 0.1591 | 0.2027 |

Interaction p-values were from logistic regression models.

Logistic regression models adjusted for sex, education level, smoking (current, former, never), alcohol consumption (current, former, never), tea (current, former, never), coffee (no/yes), BMI (≤18.5, 18.5 to < 24, ≥24 kg/m^2^), use of earphone (no/yes), tinnitus (no/yes), electric otoscopy (normal/abnormal), occupational noise exposure (no/yes), use of ototoxic medication (no/yes), hypertension (no/yes), dyslipidemia (no/yes), and corresponding interaction terms.

BMI indicates body mass index; FBG indicates fasting blood glucose.

**Table S2 Association between diabetes, duration and FBG and speech-, low/mid- and high-frequency hearing loss stratified by age group (< 65/≥65 years)**

| **Variable** | **No diabetes** | **Diabetes** | | **Duration group** | | | | | **Continuous per 1-mmol/L increment** | |
| --- | --- | --- | --- | --- | --- | --- | --- | --- | --- | --- |
|  |  |  |  | **0 to 5 years duration** | | | **> 5 years duration** | |  |  |
|  |  | OR (95% CI) | *P* value | OR (95% CI) | *P* value | | OR (95% CI) | *P* value | OR (95% CI) | *P* value |
| < 65 | 2013/2268 | 255/2268 |  | 165 | |  | 84 |  | 2267 |  |
| Speech frequency hearing loss | 1 (ref) | **1.50 (1.12, 2.00)** | 0.0059 | **1.42 (1.00, 2.01)** | | 0.0488 | **1.70 (1.06, 2.73)** | 0.0290 | **1.12 (1.05, 1.20)** | 0.0015 |
| Low/mid-frequency hearing loss | 1 (ref) | **1.45 (1.09, 1.93)** | 0.0099 | 1.28 (0.91, 1.81) | | 0.1534 | **1.88 (1.18, 3.00)** | 0.0083 | **1.12 (1.04, 1.19)** | 0.0012 |
| High-frequency hearing loss | 1 (ref) | **1.82 (1.30, 2.55)** | 0.0005 | **1.64 (1.10, 2.46)** | | 0.0158 | **2.33 (1.29, 4.22)** | 0.0051 | **1.15 (1.05, 1.25)** | 0.0021 |
| >=65 | 407/553 | 146/553 |  | 71 | |  | 72 |  | 553 |  |
| Speech frequency hearing loss | 1 (ref) | 1.24 (0.73, 2.12) | 0.4265 | 1.20 (0.59, 2.43) | | 0.6119 | 1.35 (0.65, 2.81) | 0.4202 | 1.10 (0.92, 1.32) | 0.2923 |
| Low/mid-frequency hearing loss | 1 (ref) | 0.91 (0.58, 1.42) | 0.6623 | 0.74 (0.42, 1.32) | | 0.3072 | 1.15 (0.62, 2.15) | 0.6630 | 0.99 (0.86, 1.13) | 0.8757 |
| High-frequency hearing loss | 1 (ref) | 1.19 (0.44, 3.22) | 0.7388 | 1.19 (0.32, 4.38) | | 0.7962 | 1.09 (0.29, 4.17) | 0.8979 | 0.96 (0.69, 1.32) | 0.7943 |

OR (95% CI) were from logistic regression models.

Logistic regression models adjusted for sex, education level, smoking (current, former, never), alcohol consumption (current, former, never), tea (current, former, never), coffee (no/yes), BMI (≤18.5, 18.5 to < 24, ≥24 kg/m^2^), use of earphone (no/yes), tinnitus (no/yes), electric otoscopy (normal/abnormal), occupational noise exposure (no/yes), use of ototoxic medication (no/yes), hypertension (no/yes) and dyslipidemia (no/yes).

In adults aged <65 years, the number of participants in models is 2268 (2013+255) when comparison is between diabetes and no diabetes, 2262 (2013+165+84) when comparison is between duration groups and no diabetes, 2267 when modeled FBG as a continuous variable. In adults aged ≥65 years, the number of participants in models is 553 (407+146) when comparison is between diabetes and no diabetes, 550 (407+71+72) when comparison is between duration groups and no diabetes, 553 when modeled FBG as a continuous variable. The inconsistency of the number of participants across different models is due to the missing of explanatory variable values.

BMI indicates body mass index; FBG indicates fasting blood glucose; OR indicates odds ratios; CI indicates confidence intervals.

**Table S3 Association between diabetes, duration and FBG and speech-, low/mid- and high-frequency hearing loss stratified by sex**

| **Variable** | **No diabetes** | **Diabetes** | | **Duration group** | | | | **Continuous per 1-mmol/L increment** | |
| --- | --- | --- | --- | --- | --- | --- | --- | --- | --- |
|  |  |  |  | **0 to 5 years duration** | | **> 5 years duration** | |  |  |
|  |  | OR (95% CI) | *P* value | OR (95% CI) | *P* value | OR (95% CI) | *P* value | OR (95% CI) | *P* value |
| Women | 1458/1641 | 183/1641 |  | 110 |  | 66 |  | 1640 |  |
| Speech frequency hearing loss | 1 (ref) | **1.43 (1.01, 2.05)** | 0.0472 | 1.31 (0.84, 2.02) | 0.2319 | 1.70 (0.96, 3.03) | 0.0712 | **1.11 (1.01, 1.23)** | 0.0347 |
| Low/mid-frequency hearing loss | 1 (ref) | 1.23 (0.87, 1.75) | 0.2416 | 1.06 (0.69, 1.63) | 0.7945 | 1.62 (0.92, 2.87) | 0.0976 | 1.05 (0.96, 1.16) | 0.2917 |
| High-frequency hearing loss | 1 (ref) | 1.33 (0.88, 2.00) | 0.1763 | 1.22 (0.74, 2.01) | 0.4294 | 1.53 (0.76, 3.08) | 0.2357 | 1.08 (0.96, 1.20) | 0.2034 |
| Men | 962/1180 | 218/1180 |  | 126 |  | 90 |  | 1180 |  |
| Speech frequency hearing loss | 1 (ref) | 1.39 (0.97, 2.00) | 0.0757 | 1.41 (0.90, 2.21) | 0.1378 | 1.46 (0.84, 2.55) | 0.1780 | **1.12 (1.03, 1.22)** | 0.0106 |
| Low/mid-frequency hearing loss | 1 (ref) | 1.27 (0.91, 1.78) | 0.1640 | 1.14 (0.75, 1.73) | 0.5330 | 1.56 (0.85, 2.57) | 0.0819 | **1.11 (1.03, 1.20)** | 0.0060 |
| High-frequency hearing loss | 1 (ref) | **2.55 (1.51, 4.30)** | 0.0004 | **2.28 (1.20, 4.33)** | 0.0114 | **3.39 (1.40, 8.19)** | 0.0067 | **1.22 (1.06, 1.40)** | 0.0044 |

OR (95% CI) were from logistic regression models.

Logistic regression models adjusted for age(< 65/≥65 years), education level, smoking (current, former, never), alcohol consumption (current, former, never), tea (current, former, never), coffee (no/yes), BMI (≤18.5, 18.5 to < 24, ≥24 kg/m^2^), use of earphone (no/yes), tinnitus (no/yes), electric otoscopy (normal/abnormal), occupational noise exposure (no/yes), use of ototoxic medication (no/yes), hypertension (no/yes) and dyslipidemia (no/yes).

In women, the number of participants in models is 1641 (1458+183) when comparison is between diabetes and no diabetes, 1634 (1458+110+66) when comparison is between duration groups and no diabetes, 1640 when modeled FBG as a continuous variable. In men, the number of participants in models is 1180 (962+218) when comparison is between diabetes and no diabetes, 1178 (962+126+90) when comparison is between duration groups and no diabetes, 1180 when modeled FBG as a continuous variable. The inconsistency of the number of participants across different models is due to the missing of explanatory variable values.

BMI indicates body mass index; FBG indicates fasting blood glucose; OR indicates odds ratios; CI indicates confidence intervals.

**Table S4 Association between diabetes, duration and FBG and speech-, low/mid- and high-frequency hearing loss after excluding all participants with abnormal electric otoscopy**

| **Variable** | **No diabetes** | **Diabetes** | | **Duration group** | | | | **Continuous per 1-mmol/L increment** | |
| --- | --- | --- | --- | --- | --- | --- | --- | --- | --- |
|  |  |  |  | **0 to 5 years duration** | | **> 5 years duration** | |  |  |
|  |  | OR (95% CI) | *P* value | OR (95% CI) | *P* value | OR (95% CI) | *P* value | OR (95% CI) | *P* value |
| Speech frequency hearing loss | 1 (ref) | **1.43 (1.11, 1.85)** | 0.0058 | 1.34 (0.98, 1.84) | 0.0640 | **1.64 (1.10, 2.45)** | 0.0156 | **1.12 (1.05, 1.20)** | 0.0006 |
| Low/mid-frequency hearing loss | 1 (ref) | **1.29 (1.01, 1.64)** | 0.0436 | 1.11 (0.82, 1.50) | 0.4869 | **1.66 (1.14, 2.43)** | 0.0089 | **1.10 (1.03, 1.17)** | 0.0022 |
| High-frequency hearing loss | 1 (ref) | **1.77 (1.29, 2.44)** | 0.0005 | **1.56 (1.06, 2.30)** | 0.0231 | **2.28 (1.32, 3.97)** | 0.0034 | **1.14 (1.05, 1.24)** | 0.0019 |

OR (95% CI) were from logistic regression models.

Logistic regression models adjusted for age(< 65/≥65 years), sex, education level, smoking (current, former, never), alcohol consumption (current, former, never), tea (current, former, never), coffee (no/yes), BMI (≤18.5, 18.5 to < 24, ≥24 kg/m^2^), use of earphone (no/yes), tinnitus (no/yes), occupational noise exposure (no/yes), use of ototoxic medication (no/yes), hypertension (no/yes) and dyslipidemia (no/yes).

BMI indicates body mass index; FBG indicates fasting blood glucose; OR indicates odds ratios; CI indicates confidence intervals.

**Table S5 Association between diabetes, duration and FBG and speech-, low/mid- and high-frequency hearing loss removing from the models the adjustment for tinnitus**

| **Variable** | **No diabetes** | **Diabetes** | | **Duration group** | | | | **Continuous per 1-mmol/L increment** | |
| --- | --- | --- | --- | --- | --- | --- | --- | --- | --- |
|  |  |  |  | **0 to 5 years duration** | | **> 5 years duration** | |  |  |
|  |  | OR (95% CI) | *P* value | OR (95% CI) | *P* value | OR (95% CI) | *P* value | OR (95% CI) | *P* value |
| Speech frequency hearing loss | 1 (ref) | **1.42 (1.11, 1.82)** | 0.0061 | 1.35 (0.99, 1.83) | 0.0608 | **1.60 (1.08, 2.37)** | 0.0196 | **1.12 (1.05, 1.20)** | 0.0005 |
| Low/mid-frequency hearing loss | 1 (ref) | 1.27 (1.00, 1.61) | 0.0551 | 1.10 (0.82, 1.48) | 0.5204 | **1.61 (1.11, 2.33)** | 0.0128 | **1.09 (1.03, 1.16)** | 0.0037 |
| High-frequency hearing loss | 1 (ref) | **1.73 (1.26, 2.37)** | 0.0007 | **1.56 (1.06, 2.29)** | 0.0232 | **2.10 (1.23, 3.60)** | 0.0067 | **1.13 (1.04, 1.23)** | 0.0034 |

OR (95% CI) were from logistic regression models.

Logistic regression models adjusted for age(< 65/≥65 years), sex, education level, smoking (current, former, never), alcohol consumption (current, former, never), tea (current, former, never), coffee (no/yes), BMI (≤18.5, 18.5 to < 24, ≥24 kg/m^2^), use of earphone (no/yes), electric otoscopy (normal/abnormal), occupational noise exposure (no/yes), use of ototoxic medication (no/yes), hypertension (no/yes) and dyslipidemia (no/yes).

BMI indicates body mass index; FBG indicates fasting blood glucose; OR indicates odds ratios; CI indicates confidence intervals.

**Table S6 Association between diabetes, duration and FBG and speech-, low/mid- and high-frequency hearing loss adjusting for** **menopausal status (no/yes) additionally in women**

| **Variable** | **No diabetes** | **Diabetes** | | **Duration group** | | | | **Continuous per 1-mmol/L increment** | |
| --- | --- | --- | --- | --- | --- | --- | --- | --- | --- |
|  |  |  |  | **0 to 5 years duration** | | **> 5 years duration** | |  |  |
|  |  | OR (95% CI) | *P* value | OR (95% CI) | *P* value | OR (95% CI) | *P* value | OR (95% CI) | *P* value |
| Speech frequency hearing loss | 1 (ref) | 1.42 (0.99, 2.04) | 0.0581 | 1.34 (0.85, 2.10) | 0.2078 | 1.53 (0.85, 2.74) | 0.1536 | **1.12 (1.01, 1.24)** | 0.0290 |
| Low/mid-frequency hearing loss | 1 (ref) | 1.21 (0.85, 1.73) | 0.2782 | 1.07 (0.69, 1.65) | 0.7756 | 1.47 (0.83, 2.61) | 0.1897 | 1.06 (0.96, 1.17) | 0.2873 |
| High-frequency hearing loss | 1 (ref) | 1.31 (0.86, 2.02) | 0.2131 | 1.30 (0.77, 2.20) | 0.3894 | 1.38 (0.67, 2.85) | 0.3264 | 1.07 (0.95, 1.20) | 0.2477 |

OR (95% CI) were from logistic regression models.

Logistic regression models adjusted for age(< 65/≥65 years), sex, education level, smoking (current, former, never), alcohol consumption (current, former, never), tea (current, former, never), coffee (no/yes), BMI (≤18.5, 18.5 to < 24, ≥24 kg/m^2^), use of earphone (no/yes), electric otoscopy (normal/abnormal), occupational noise exposure (no/yes), use of ototoxic medication (no/yes), hypertension (no/yes) and dyslipidemia (no/yes) and menopausal status (no/yes).

BMI indicates body mass index; FBG indicates fasting blood glucose; OR indicates odds ratios; CI indicates confidence intervals.

**Table S7 The associations of diabetes and other cardiometabolic diseases with speech-, low/mid-, and high- frequency hearing loss**

| **Variable** | **Speech frequency hearing loss** | | **High-frequency hearing loss** | |
| --- | --- | --- | --- | --- |
|  | OR (95% CI) | *P* | OR (95% CI) | *P* |
| Diabetes | 1.44 (1.12, 1.86) | 0.0046 | 1.75 (1.28, 2.41) | 0.0005 |
| Overweight/Obesity | 1.35 (1.13, 1.62) | 0.0360 | 1.19 (0.98, 1.45) | 0.1307 |
| Hypertension | 1.44 (1.20, 1.73) | <.0001 | 1.74 (1.43, 2.12) | <.0001 |
| Dyslipidemia | 1.18 (0.95, 1.46) | 0.1372 | 1.39 (1.09, 1.79) | 0.0091 |

OR (95% CI) were from logistic regression models.

Logistic regression models adjusted for age(< 65/≥65 years), sex, education level, smoking (current, former, never), alcohol consumption (current, former, never), tea (current, former, never), coffee (no/yes), use of earphone (no/yes), electric otoscopy (normal/abnormal), occupational noise exposure (no/yes), use of ototoxic medication (no/yes), diabetes (no/yes), overweight/obesity (BMI 18.5 to < 24/≥24 kg/m^2^), hypertension (no/yes) and dyslipidemia (no/yes), as appropriate.

BMI indicates body mass index; FBG indicates fasting blood glucose; OR indicates odds ratios; CI indicates confidence intervals.
